# MRE11 stability is regulated by CK2-dependent interaction with R2TP complex

**DOI:** 10.1038/onc.2017.99

**Published:** 2017-04-24

**Authors:** P von Morgen, K Burdova, T G Flower, N J O'Reilly, S J Boulton, S J Smerdon, L Macurek, Z Hořejší

**Affiliations:** 1 Department of Cancer Cell Biology, Institute of Molecular Genetics of the ASCR, Prague, Czech Republic; 2 Faculty of Science, Charles University, Prague, Czech Republic; 3 Structural Biology of DNA-damage Signalling Laboratory, The Francis Crick Institute, London,UK; 4 Peptide Chemistry, The Francis Crick Institute, London, UK; 5 DSB Repair Metabolism Laboratory, The Francis Crick Institute, London, UK; 6 Centre for Molecular Oncology, Barts Cancer Institute, Queen Mary University of London, John Vane Centre, Charterhouse Square, London, UK

## Abstract

The MRN (MRE11–RAD50–NBS1) complex is essential for repair of DNA double-strand breaks and stalled replication forks. Mutations of the MRN complex subunit MRE11 cause the hereditary cancer-susceptibility disease ataxia-telangiectasia-like disorder (ATLD). Here we show that MRE11 directly interacts with PIH1D1, a subunit of heat-shock protein 90 cochaperone R2TP complex, which is required for the assembly of large protein complexes, such as RNA polymerase II, small nucleolar ribonucleoproteins and mammalian target of rapamycin complex 1. The MRE11-PIH1D1 interaction is dependent on casein kinase 2 (CK2) phosphorylation of two acidic sequences within the MRE11 C terminus containing serines 558/561 and 688/689. Conversely, the PIH1D1 phospho-binding domain PIH-N is required for association with MRE11 phosphorylated by CK2. Consistent with these findings, depletion of PIH1D1 resulted in MRE11 destabilization and affected DNA-damage repair processes dependent on MRE11. Additionally, mutations of serines 688/689, which abolish PIH1D1 binding, also resulted in decreased MRE11 stability. As depletion of R2TP frequently leads to instability of its substrates and as truncation mutation of MRE11 lacking serines 688/689 leads to decreased levels of the MRN complex both in ATLD patients and an ATLD mouse model, our results suggest that the MRN complex is a novel R2TP complex substrate and that their interaction is regulated by CK2 phosphorylation.

## Introduction

Genome instability is one of the hallmarks of cancer cells and is frequently associated with mutations in genes involved in the DNA-damage response (DDR). *NBS1* and *MRE11* genes code for proteins essential for recognition and repair of damaged DNA and their mutation predisposes to hereditary cancer-prone Nijmegen breakage syndrome (NBS) and ataxia-telangiectasia-like disorder (ATLD), respectively.^1, 2^ Moreover, mutations of both genes have been found in patients with breast and colon cancer.^3, 4, 5, 6^ MRE11, NBS1 and RAD50 form the MRN complex that allows recognition of DNA double-strand breaks, activates two major DDR kinases ataxia-telangiectasia mutated (ATM) and ataxia-telangiectasia and Rad3 related (ATR), stabilizes ends of broken DNA and facilitates DNA repair by homologous recombination (HR) and non-homologous end-joining.^7, 8, 9, 10^

**Figure 1:**
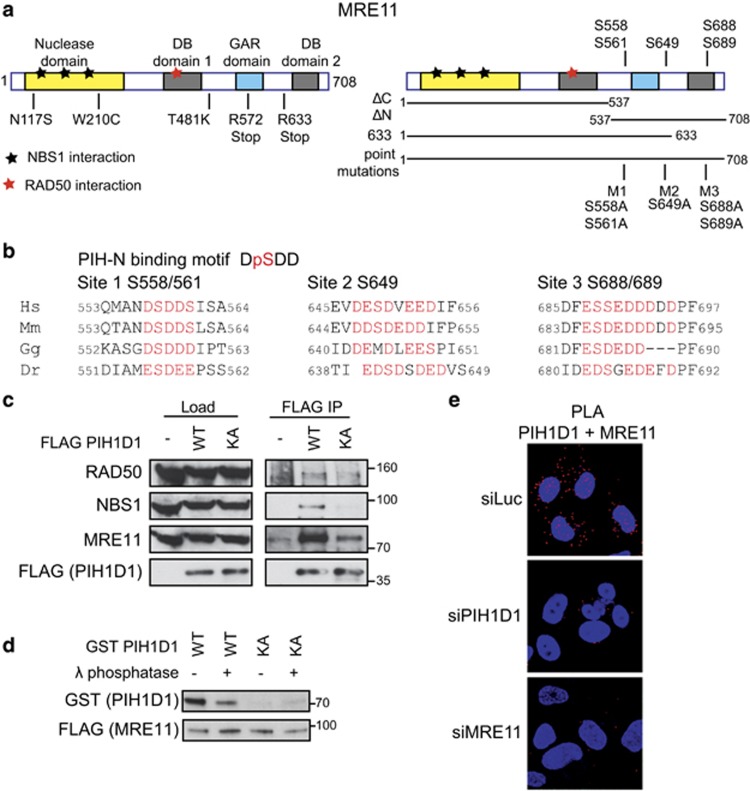
PIH1D1 interacts with MRN complex. (**a**) Schematic representation of human MRE11 comprising of a nuclease domain, two DNA-binding domains (DB1 and DB2) and glycine–arginine-rich (GAR) domain. Positions of ATLD mutations (left panel) and putative PIH-N phospho-binding sites (right panel) are indicated. Bars below the right panel represent the MRE11 constructs used in this study. (**b**) Conservation of the PIH-N consensus motif in the C terminus of MRE11. Acidic amino acids and serines that form potential PIH-N-binding sites are displayed in red. (**c**) FLAG PIH1D1 WT interacts with MRN complex components. HEK293T cells transfected with FLAG PIH1D1 WT or phospho-binding mutant PIH1D1 KA were lysed in immunoprecipitation (IP) buffer (50 mM Tris-HCl (pH 7.5), 150 mM NaCl, 1% Triton X-100, 1 mM EDTA, 2.5 mM EGTA, 10% (v/v) glycerol supplemented with cOmplete EDTA-free protease inhibitor, PhosSTOP phosphatase inhibitor (Sigma-Aldrich, St Louis, MO, USA) and EtBr (50 μg/ml)) and sonicated 3 × 10s. Cleared cell extracts were incubated with anti-FLAG M2 affinity gel (Sigma-Aldrich) for 2 h. Beads were washed 4x with IP buffer, boiled in 2xLSB buffer (100 mM Tris (pH 6.8), 200 mM dithiothreitol (DTT), 4% sodium dodecyl sulfate (SDS), 0,2% bromophenol blue, 20% (v/v) glycerol) and bound proteins were detected by immunoblotting using antibodies against RAD50 (Abcam, Cambridge, UK; ab119708), MRE11 (Cell Signaling, Danvers, MA, USA; no. 4895) and NBS1 (Cell Signaling; no. 3002). (**d**) MRE11–PIH1D1 interaction depends on MRE11 phosphorylation and on the PIH-N domain of PIH1D1. FLAG MRE11 was purified from HEK293T cells using anti-FLAG M2 affinity gel. Beads were washed with IP buffer supplemented with 1 m NaCl, treated or not with λ phosphatase for 30 min at 30 °C and MRE11 was eluted with 3 × FLAG peptide (30 μg; Sigma-Aldrich). GST PIH1D1 WT and phospho-binding mutant GST PIH1D1 KA were purified from BL21 *Escherichia coli* and pull-down with purified MRE11 was performed as described previously.^20^ (**e**) MRE11 and PIH1D1 interact *in vivo*. U2OS cells grown on coverslips were transfected with 40 nm siRNA targeting luciferase (5′-CGUACGCGGAAUACUUCGA-3′ Sigma), PIH1D1 (1:1 mixture of 5′-GAAUGGAAAUGUAGUCUUA-3′ and 5′-GAGAAGAGGCUGCUGGCUU-3′ in the untranslated region of PIH1D1 as described^20^) or MRE11 (5′-GAGCAUAACUCCAUAAGUA-3′ in the untranslated region of MRE11 mRNA) with Lipofectamine RNAiMAX reagent (Thermo Fisher Scientific, Waltham, MA, USA) according to the manufacturer's instructions. Cells were permeabilized with 0.2% Triton X-100 for 5 min at room temperature and proximity ligation assay (PLA) was performed using MRE11 (Cell Signaling; no. 4895), PIH1D1 (Abcam; ab57512) antibodies and Duolink reagent (Sigma-Aldrich) according to the manufacturer's protocol. Red signal is proximity ligations assay (PLA) staining, and 4',6-diamidino-2-phenylindole (DAPI) is in blue. Representative image is shown.

ATLD patients develop cerebellar ataxia and their cells exhibit chromosomal instability, radiosensitivity and DNA-damage-dependent checkpoint defects.^11^ Mutations in the N-terminal part of MRE11, containing nuclease domain important for initiation of HR, cause mainly structural and functional defects of the protein.^10^ Several of these mutations prevent interactions between MRE11, NBS1 and RAD50 and are associated with decreased levels of MRN complex.^12^ The MRE11 C-terminal part contains two potential DNA-binding domains and a glycine–arginine-rich motif, involved in the regulation of MRE11 nuclease activity and DNA binding (Figure 1a).^13, 14^ Moreover, phosphorylation of serines 676 and 678 by ATM and ribosomal S6 kinase and interaction of the last 13 amino acids (aa) of MRE11 C terminus with CDK2 affect processing of damaged DNA ends.^15, 16, 17^ In contrast to the ATLD single-point mutations, MRE11 truncated at R633 (known as ATLD1) binds NBS1 and RAD50. Interestingly, the level of MRN complex in ATLD1 patient cells is also decreased. While*Mre11*^*ATLD1/ATLD1*^ mice completely recapitulate the checkpoint phenotypes seen in ATLD patients including decreased level of MRN complex, it is currently unclear why the MRN complex becomes unstable in the ATLD1 human and *Mre11*^*ATLD1/ATLD1*^ mice cells.^18^

The C terminus of MRE11 encompasses several conserved acidic motifs that are predicted consensus phosphorylation sites of casein kinase 2 (CK2) and resemble binding sites for the PIH1D1 subunit of the heat-shock protein 90 (HSP90) cochaperone complex R2TP (Figure 1b).^19, 20^ The R2TP complex is involved in the assembly of large protein complexes such as small nucleolar ribonucleoproteins, RNA polymerase II and complexes comprising phosphatidylinositol 3 kinase-related kinases, including major DDR kinases ATM, ATR and DNA-dependent protein kinase (DNA-PK).^21, 22, 23, 24^ It contains two subunits specific for the complex—PIH1D1, which possesses a PIH-N domain involved in the recognition of R2TP complex substrates—and RPAP3, which interacts with HSP90 by its tetratricopeptide domains. In addition, the complex comprises two ATPases, RUVBL1 and RUVBL2, that are associated with many other cellular complexes.^19, 20, 24, 25, 26, 27^ Depletion of the R2TP complex frequently leads to instability of its substrates, including PI3Ks and RNA polymerase II.^21, 22, 24, 28^

Here we show that PIH1D1 binds directly to two CK2-phosphorylated sites within the MRE11 C terminus. We further demonstrate that depletion of PIH1D1 resulted in MRE11 destabilization and that mutations of PIH1D1-binding sites in MRE11 C terminus decrease MRE11 stability. In addition, depletion of PIH1D1 impaired DDR processes regulated by MRN complex. Taken together, we provide an insight into the molecular basis of MRN complex instability in ATLD1 patients and reveal a new mode of regulation of MRN complex activity by CK2 and the HSP90 cochaperone R2TP complex.

## Results and discussion

### PIH1D1 interacts directly with MRE11 in a phospho-dependent manner

We and others have previously shown that PIH1D1 binds acidic sequence DpSDD phosphorylated by CK2.^19, 20^ It has been suggested that PIH-N domain may bind to this conserved motif present in MRE11 C terminus (serine 558).^19^ The MRE11 C terminus also contains another two highly acidic regions conserved in mammals, which may be potentially recognized by PIH-N domain (Figure 1b). To determine if R2TP complex is involved in MRN complex assembly, we first investigated whether PIH1D1 can interact with MRE11 and if the interaction requires PIH1D1 phospho-binding ability. Overexpressed FLAG-tagged wild-type PIH1D1 (FLAG PIH1D1 WT) co-immunoprecipitated with components of MRN complex, whereas PIH1D1 K64A mutation that abolishes phospho-binding (FLAG PIH1D1 KA) failed to do so^20^ (Figure 1c). Furthermore, FLAG MRE11 WT purified from whole-cell extract pulled down recombinant wild-type GST-tagged PIH1D1 (GST PIH1D1 WT) and this interaction was abolished either by dephosphorylation of MRE11 by λ phosphatase or by the PIH1D1 K64A mutation (Figure 1d). We further confirmed the interaction between MRE11 and PIH1D1 both in the cytoplasm and the nucleus *in vivo* by a proximity ligation assay (Figure 1e). Based on these data, we conclude that PIH1D1 binds MRE11 directly *in vitro* and *in vivo* and that the interaction depends on phosphorylation of MRE11 and an intact PIH1D1 phospho-binding domain PIH-N. As PIH1D1 has only been shown to be exclusively active as a part of the R2TP complex, our data suggest that MRE11 and possibly the whole MRN complex may be a substrate of this HSP90 cochaperone.

### PIH1D1 interacts with phosphorylated MRE11 serine 558/561 and S688/689

**Figure 2:**
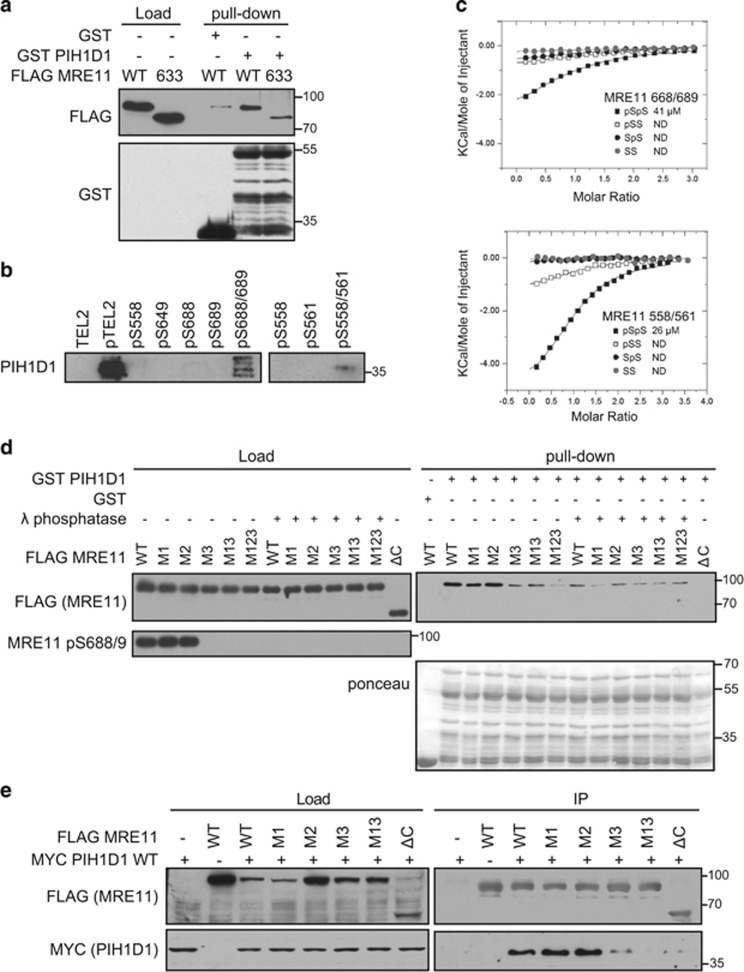
Phosphorylated serine 558/561 and 688/689 of MRE11 interact directly with PIH1D1. (**a**) MRE11 ATLD1 truncation reduces binding of PIH1D1. GST or GST PIH1D1 WT were bound to glutathione sepharose beads (GenScript, Piscataway, NJ, USA) for 2 h at 4 °C, washed with TBS-Tween (150 mM NaCl, 3 mM KCl, 25 mM Tris-HCl (pH 7.2), 10% glycerol) and incubated with 90μl of eluted FLAG MRE11 WT or FLAG MRE11 truncated at residue 633 for 1h at 4 °C. The beads were washed 4x with TBS-Tween and boiled in 2xLSB buffer (100 mm Tris (pH 6.8), 200 mm dithiothreitol (DTT), 4% sodium dodecyl sulfate (SDS), 0,2% bromophenol blue, 20% (v/v) glycerol). Bound proteins were analyzed by immunoblotting. Recombinant full-length GST PIH1D1 is at mark 55 kDa, and other bands are degradation products. (**b**) Biotinylated peptides containing a double phosphorylation of serine 558/561 or 688/681 pull-down PIH1D1. Biotinylated peptides were synthesized by the Francis Crick Institute Peptide Chemistry facility and peptide pull-down from HeLa nuclear extract (4 mg/ml; Ipratech, Mons, Belgium) was performed as described previously.^20^ Bound PIH1D1 was analyzed by immunoblotting. Phosphorylated and non-phosphorylated TEL2 peptides were used as positive and negative controls, respectively. (**c**) Isothermal titration calorimetric (ITC) analysis of MRE11 binding (see Supplementary Tables S2a and S2b for sequences) to PIH1D1 1–180. Purification of PIH1D1 1–180 PIH-N domain fragment and ITC was performed as described previously. (**d**) Phosphorylation of serine 688/689 is required for interaction between MRE11 and PIH1D1. GST or GST PIH1D1 WT were incubated with purified FLAG MRE11 WT or mutants M1, M2, M3, M13, M123 and interaction was assessed by immunoblotting against FLAG. Where indicated, purified MRE11 was treated with λ phosphatase prior the pull-down assay. Staining with MRE11 pS688/9 antibody was used as control of efficient dephosphorylation. Recombinant full-length GST PIH1D1 is at mark 55 kDa, and other bands are degradation products. (**e**) Interaction between MRE11 and PIH1D1 in cells depends on serine 688/689. HCT116 cells were co-transfected with plasmids coding MYC PIH1D1 and FLAG MRE11 WT, M1, M2, M3, M13 or MRE11 ΔC mutants. Cell extracts were incubated with anti-FLAG M2 affinity gel and immunoprecipitated proteins were analyzed with MYC (GeneTex, Zeeland, MI, USA; GTX115046) and FLAG antibodies.

Truncated MRE11 in ATLD1 preserves serine 558, but at the same time it is missing the two other acidic sites. To test whether the ATLD1 mutant is able to bind PIH1D1, we performed pull-down assay with recombinant GST PIH1D1 WT and FLAG MRE11 WT or MRE11 ATLD1 truncation (FLAG MRE11 633) purified from whole-cell extracts. Importantly, the FLAG MRE11 633 interaction with PIH1D1 is greatly reduced (Figure 2a), suggesting that the DpSDD motif may not be the main or the only site responsible for the interaction. To identify which of the acidic motifs further down within the C terminus participates in the interaction, we performed peptide pull-downs with biotinylated peptides coupled to streptavidin beads from cell extract. The peptides comprise phosphorylated and non-phosphorylated MRE11 sequences, potentially able to bind PIH1D1 (Supplementary Table S2a). We observed that the DpSDD motif-containing peptide could bind PIH1D1, but only if both serines 558 and 561 within this peptide were phosphorylated. The second site, containing a motif similar to DSDD (serine 649), did not bind PIH1D1, whereas the third acidic sequence could bind PIH1D1, but only if both serines 688 and 689 were phosphorylated (Figure 2b). We confirmed these results by an isothermal titration calorimetric assay (Figure 2c and Supplementary Table S2b), in which only the double phosphopeptides interacted with PIH1D1 N-terminal PIH-N domain fragment (aa 51–180). Although the observed affinities are in both cases ~3–5-fold weaker (26 μM for serine 558/561 and 41 μM for serine 688/689) than interaction between PIH-N and its substrate TEL2 (9.8 μM; Supplementary Table S2b), the presence of two sites able to bind PIH1D1 may increase stability of the interaction. Alternatively, other subunits of the R2TP complex may bind to other MRE11-interacting partners (such as NBS1 or RAD50), which would result in overall stronger binding.

To determine which of the two sites is the major binding site, we performed pull-down assays with GST PIH1D1 and FLAG MRE11 WT, the N-terminal part of MRE11 lacking all potential PIH1D1-binding sites (FLAG MRE11ΔC, aa 1–537) or MRE11 with serine 558/561 (M1), serine 649 (M2), serine 688/689 (M3) or their combinations mutated to alanine (FLAG MRE11 M1, M2, M3, M13 and M123). While mutation M2 had no appreciable effect on PIH1D1 binding and M1 slightly reduced interaction with PIH1D1, mutation M3, its combination with M1 (M13) and mutation of all three sites (M123) greatly reduced the interaction with PIH1D1. The interaction of FLAG MRE11 WT, M2 and M1 with PIH1D1 was abolished by λ phosphatase treatment of the purified MRE11 prior the pull-down (Figure 2d). We have confirmed these results by co-immunoprecipitating FLAG MRE11 WT, M1, M2, M3, M13 and ΔC with MYC PIH1D1 (Figure 2e). Our data suggest that the interaction between MRE11 and PIH1D1 depends on phosphorylated serine 688/689 and to some extent also on phosphorylated serine 558/561 of MRE11. Given that the binding site comprising serine 688/689 differs from the DpSDD PIH1D1-binding motif, we have identified a new target sequence of PIH-N domain.

### MRE11 serines 558/561 and 688/689 are phosphorylated by CK2

**Figure 3:**
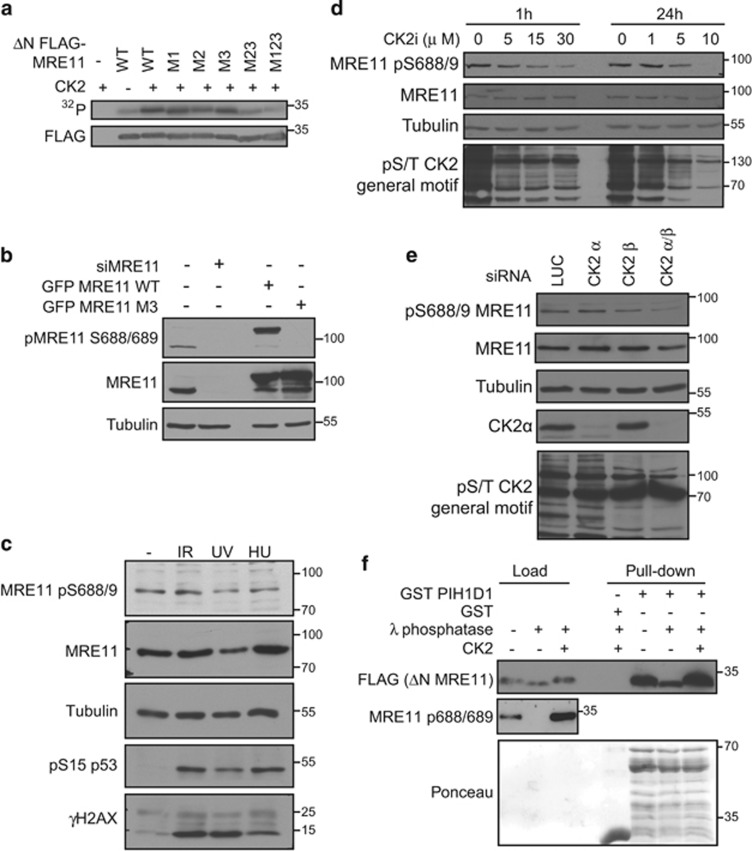
Serines 558/561 and 688/689 are phosphorylated by CK2. (**a**) CK2 phosphorylates MRE11 *in vitro*. FLAG MRE11FLAG MRE11 ΔN WT or M1, M2, M3, M23 and M123 proteins were immunoprecipitated from HEK293T cells using anti-FLAG M2 affinity gel. Beads were washed with 1m NaCl to eliminate contamination with protein kinases and incubated with 10 U of CK2 holoenzyme complex (α2/β Biaffin GmbH, Kassel, Germany) in 30 μl of kinase buffer (20 mM Tris-HCl (pH 8.0); 50 mM KCl; 10 mM MgCl_2_) supplemented with 33 μM ATP and [γ-^32^P]ATP (5 μCi) and incubated for 30 min at 37 °C. Reaction was stopped by the addition of 4 × Laemmli buffer (200 mM Tris (pH 6.8), 400 mM dithiothreitol (DTT), 8% sodium dodecyl sulfate (SDS), 0.4% bromophenol blue, 40% (v/v) glycerol) and phosphorylation of MRE11 was detected by autoradiography. Amount of the FLAG MRE11 was determined by immunoblotting. (**b**) MRE11 pS688/689 antibody specifically recognizes phosphorylated MRE11. We raised a rabbit polyclonal antibody against synthetic VSKGVDFEpSpSEDDDDD peptide, where pSpS represent phosphorylated serines corresponding to pS688/9 of human MRE11 and performed a two-step affinity purification using non-phosphorylated and phosphorylated peptide (Davids Biotechnologie GmbH, Regensburg, Germany). U2OS cells were transfected with siRNA against MRE11 (siMRE11) or with plasmids coding for GFP MRE11 WT or GFP MRE11 S688/9A. Whole-cell lysates were analyzed by immunoblotting with indicated antibodies. Antibody against tubulin (GeneTex; GTX112141) was used as a loading control. (**c**) Phosphorylation of MRE11 on serine 688/689 does not change in the presence of genotoxic stress. DNA damage was induced in U2OS cells by exposure to ionizing radiation (10 Gy 1 h, generated by X-ray instrument T-200; Wolf-Medizintechnik, St Gangloff, Germany), UVC (10 J/m^2^ for 1 h) or hydroxy urea (2 mM for 16 h) and whole-cell lysates were analyzed by immunoblotting. (**d**) CK2 inhibition decreases phosphorylation of MRE11 S688/689. U2OS cells were treated with dimethyl sulfoxide (DMSO) or CK2 inhibitor (5–30 μm, CX-4945; MedChemExpress, Monmouth Junction, NJ, USA) for 1 or 24 h and whole-cell lysates were probed with indicated antibodies. Antibody recognizing a general phospho-CK2 substrate (Cell Signaling; no. 8738) was used as the control of CK2i efficiency. (**e**) CK2 knockdown decreases phosphorylation of MRE11 S688/689. U2OS cells were treated with siRNA targeting luciferase (siLUC) and siRNA targeting CK2 subunit α, β or both for 4 days. Whole-cell lysates were probed with indicated antibodies. Antibody recognizing CK2α was from GeneTex (GTX107897). (**f**) CK2 promotes the interaction between PIH1D1 and MRE11. Purified FLAG MRE11 ΔN was dephosphorylated with λ phosphatase and rephosphorylated by recombinant CK2 as indicated and incubated with GST or GST PIH1D1. Bound proteins were analyzed by immunoblotting. Recombinant full-length GST PIH1D1 is at mark 55 kDa, and other bands are degradation products.

We have previously shown that the PIH1D1-binding motif DpSDD is phosphorylated by CK2.^19, 20^ As serines 561, 688 and 689 are also predicted consensus sequences of CK2, we tested whether these sites are indeed phosphorylated by CK2. *In vitro* kinase assays revealed that MRE11 C terminus can be phosphorylated by recombinant CK2 and that this phosphorylation is abolished by mutation of all five serines (558, 561, 649, 688 and 689) to alanine (Figure 3a). Subsequently, we raised an antibody against a synthetic peptide phosphorylated on serine S688/689 and validated its specificity on endogenous and overexpressed MRE11 by western blot and immunofluorescence. On western blot, the antibody recognizes a band of the correct molecular weight of endogenous MRE11 and, crucially, the signal disappears after treatment with MRE11 small interfering RNA (siRNA) (siMRE11) (Figure 3b). Consistent with the predominantly nuclear localization of MRE11, the antibody stains cell nuclei in non-transfected cells and in both the nuclei and cytoplasm in cells overexpressing MYC MRE11 WT (Supplementary Figure S3). In contrast, the antibody does not recognize overexpressed MYC and GFP MRE11 M3 on western blot and immunofluorescence (Figure 3b and Supplementary Figure S3). The overall level of phosphorylated serine 688/689 did not change after various DNA-damage treatments, consistent with the constitutive activity of CK2 (Figure 3c). To confirm that serines 688 and 689 are phosphorylated by CK2 *in vivo*, we treated U2OS cells with a CK2 inhibitor or siRNA targeting CK2 subunits α and β. Western blot analysis of cell lysates revealed that inhibition of CK2 or downregulation of both CK2 subunits reduces phosphorylation of serine 688/689 to an extent comparable to other CK2 substrates (Figures 3d and e). To further confirm that CK2 phosphorylation of serine 558/561 and serine 688/689 can promote the interaction between MRE11 and PIH1D1, we performed pull-down assays with recombinant GST PIH1D1 and MRE11 C-terminal part (FLAG MRE11ΔN, aa 537–708) purified from whole-cell extract (Figure 3f), treated or untreated with λ phosphatase. Dephosphorylation of the MRE11 C terminus abolished the interaction, whereas rephosphorylation of the ΔN construct by recombinant CK2 rescued the interaction. Based on these results, we conclude that the PIH1D1/MRE11 interaction is dependent on CK2 phosphorylation. MRE11 is phosphorylated on serine 688/689 in the cytoplasm and nucleus, which is in accordance with our proximity ligation assay results showing MRE11 and PIH1D1 interaction in both cellular compartments.

### The R2TP complex is important for DDR and for MRE11 stability

**Figure 4:**
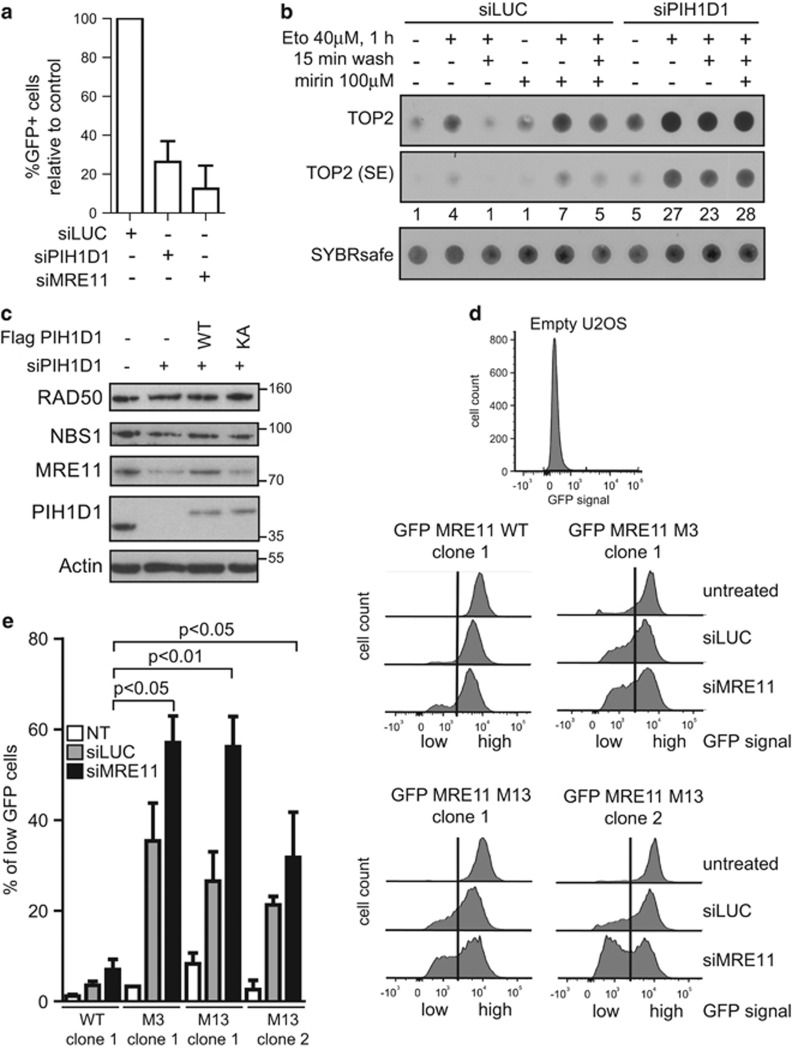
The interaction of MRE11 with PIH1D1 is important for MRE11 stability. (**a**) Depletion of PIH1D1 negatively affects HR. U2OS-DR-GFP cells^32^ were transfected every 2 days with 40nm siRNA targeting either luciferase, PIH1D1, MRE11 or combinations. For expression of ISceI, cells were transfected with pCBASce and efficiency of homologous repair was determined after 48 h as the percentage of GFP-positive cells by flow cytometry (*n*=4). Bars show the normalized mean with s.d. (**b**) Depletion of PIH1D1 impairs removal of TOP2 adducts from DNA. Formation of TOP2-DNA adducts was determined by modified RADAR assay as described.^37^ U2OS cells were transfected every 2 days for 8 days with 40 nM of siLUC or a mixture of two siRNAs targeting PIH1D1, treated with 40 μm etoposide for 1 h in the presence or absence of 100 μm mirin, washed for 15 min where indicated and lysed in 400 μl RLT buffer (Qiagen, Hilden, Germany). DNA was precipitated by 100 μl of sodium acetate (3 m, pH 5.2) and 1 ml ethanol and centrifuged at 20 000 *g*. Pellet was resuspended in 8mm NaOH and DNA concentration was determined by Hoechst 33258 in a TNE buffer (2 m NaCl, 1 mM EDTA, 50 mM Tris (pH 7.5)) using Envision 2104 multilabel reader (Perkin-Elmer, Waltham, MA, USA). DNA (200 ng) was incubated with SYBR safe in (50 mM Tris (pH 7.5), 150 mM NaCl) and slot blotted onto nitrocellulose and detected by Quantum Imaging System (Vilbert Lourmat, Marne-la-Vallée, France). Membrane was incubated with topoIIα antibody (Santa Cruz; sc-365918), signal was quantified with ImageJ and normalized to the amount of DNA. (**c**) Depletion of PIH1D1 results in destabilization of MRE11. RPE cells reconstituted with empty plasmid, FLAG PIH1D1 WT or PIH1D1 KA (as described previously^20^), were treated with siRNA targeting luciferase or the 3'-untranslated region (UTR) of PIH1D1 for 10 days. Whole-cell lysates were analyzed by immunoblotting. (**d**) Mutation of serine 558/561 together with 688/69 leads to destabilization of MRE11. Individual clones of U2OS cells stably transfected with GFP MRE11 WT, M3 and M13 were transfected with siRNA targeting luciferase (siLUC), 3'-UTR of MRE11 (siMRE11) or left untreated. siRNA was retransfected every 2 days and cells were grown for 12 days. Cells were collected and incubated with Hoechst 33258. GFP signal was analyzed in Hoechst-negative cells by flow cytometry using BD LSR II (BD Biosciences, San Jose, CA, USA). Shown are representative plots. The vertical line in the plots indicates the gating; cells with GFP intensity left from the vertical line are considered to have low amounts of GFP MRE11; and cells right from the vertical line are considered to have high GFP MRE11. (**e**) The percentage of cells from (**d**) that have low GFP MRE11 was quantified using the GraphPad (La Jolla, CA, USA) Prism software (*n*=3). Bars show mean±s.d., and statistical significance was determined with the Student’s *t*-test. Variance between the samples was equal (*F*-test).

We and others have shown that R2TP complex regulates assembly and stability of ATM and ATR.^22, 29, 30^ Accordingly, we have previously shown that depletion of PIH1D1 leads to decreased phosphorylation and protein levels of p53 after γ-irradiation.^20^ To see if PIH1D1 affects processes that are directly regulated by MRN complex, we assessed efficiency of HR in U2OS/DR-GFP cells treated with control siRNA (siLUC), PIH1D1 (siPIH1D1) and MRE11 (siMRE11) siRNA.^31, 32^ Similarly to siMRE11 treatment, depletion of PIH1D1 strongly decreased HR (Figure 4a), indicating that PIH1D1 is involved in the regulation of protein complexes required for HR, such as MRN complex. To investigate whether downregulation of PIH1D1 affects other processes directly regulated by MRE11, we assayed the removal of topoisomerase 2 (TOP2) adducts from DNA in the presence and absence of MRE11 inhibitor mirin, siPIH1D1 and siLUC by a slot-blot experiment (Figure 4b).^33, 34, 35^ Treatment of cells with etoposide resulted in increased levels of TOP2 covalently bound to DNA, which was efficiently removed 15 min after the etoposide was washed off the cells. Incubation of cells with mirin led to further increased levels of TOP2 covalently bound to DNA in the presence of etoposide and TOP2 remained bound to DNA after removal of etoposide. Depletion of PIH1D1 showed increased basal loading of TOP2 on DNA, probably reflecting accumulation of endogenous DNA damage during siPIH1D1 treatment (Figure 4b). Importantly, similar to the treatment with mirin, PIH1D1 depletion strongly reduced removal of TOP2 adducts induced by etoposide. These results suggest that PIH1D1 indeed affects function of the MRN complex, although we cannot exclude that some of the observed phenotypes are also due to PIH1D1 function in assembly of other complexes.

Next, we investigated whether the R2TP complex affects the stability of MRN complex subunits. In our previous work, reconstitution of murine embryonic fibroblasts with a mutant R2TP complex cochaperone Tel2, unable to bind PIH1D1, exhibited decreased levels of phosphatidylinositol 3 kinase-related kinases only after 11 days after endogenous Tel2 knockout. Hence, to determine the importance of the R2TP complex for MRE11 stability, we treated RPE cells reconstituted with empty vector, FLAG PIH1D1 WT or FLAG PIH1D1 KA with siRNA targeting 3'-untranslated region of PIH1D1 mRNA (siPIH1D1) for 10 days. The treatment resulted in major decrease of MRE11 levels and slight reduction of NBS1 level in cells reconstituted with empty vector and FLAG PIH1D1 KA, whereas reconstitution of cells with FLAG PIH1D1 WT restored MRE11 and NBS1 levels to levels of RPE cells transfected with empty vector treated with siLUC (Figure 4c). To confirm that PIH1D1 has an impact on stability of MRE11 in other cell types, we have treated U2OS cells with siLUC or siPIH1D1 for 7 days and HCT116 cells for 10 days. The treatment with siPIH1D1 led to decreased levels of MRE11 and slight decrease of RAD50 and NBS1 in contrast to treatment with siLUC (Supplementary Figure S4a). As downregulation of MRE11 by siRNA leads to prominent instability of NBS1 and RAD50 (Supplementary Figure S4b), we hypothesize that prolonged treatment of the cells with siPIH1D1 would ultimately lead to much more pronounced instability of RAD50 and NBS1. However, as prolonged siPIH1D1 treatment is lethal, we were unable to prove this hypothesis. We have noted that the effect of siPIH1D1 on MRE11 stability requires highly efficient knockdown of PIH1D1 and we suppose that even a small level of PIH1D1 in the cells is sufficient to promote assembly of MRN complex or maintaining of MRE11 stability. Therefore, a faster major effect on stability of all MRN complex components would require PIH1D1 levels reaching zero. However, we have not been able to knockout *PIH1D1* by CRISPR because *PIH1D1* is an essential gene in human cells and its knockout leads to cellular death.^36^

Next, we investigated whether mutation of MRE11 interaction sites with PIH1D1 also leads to destabilization of MRE11. We established U2OS cell lines stably expressing GFP MRE11 WT, M1, M3 and M13, and selected two clones for MRE11 WT and M13 and one clone for MRE11 M1 and M3 with comparable expression levels of exogenous MRE11 (Supplementary Figure S4c). To verify that the constructs are functional in cells *in vivo*, we tested their ability to be recruited to the site of the DNA damage and to repair damaged DNA. Experiments with laser irradiation-induced damage showed that all proteins are indeed recruited to sites of DNA damage (Supplementary Figure S4d). In addition, GFP MRE11 WT, M3 and M13 are able to rescue formation of RPA foci in the S-phase cells after camptothecin treatment in cells where endogenous MRE11 has been downregulated by siRNA (Supplementary Figure S4e). Interestingly, we observed a significant decrease of RPA foci in cells reconstituted with GFP MRE11 M1. Our results confirm that we have generated a relevant model system to study the effect of the selected mutations on the physiological functions and stability of MRE11. Furthermore, these results show that serine mutations do not affect MRE11 binding to the site of the damage and suggest that phosphorylation of serine 558/561 and 688/689 may regulate MRE11 in a distinct manner as their mutation has a different outcome on the processing of broken DNA ends.

As MRE11 forms dimer and the presence of endogenous MRE11 could affect the stability of the GFP constructs, we treated the U2OS cells reconstituted with the GFP MRE11 constructs with siLUC or siMRE11 for 12 or 17 days prior the assessment of MRE11 stability. Fluorescence-activated cell sorting analysis revealed that cells untreated with siRNA maintain stable levels of all GFP constructs. After treatment with control siLUC, the levels of GFP signal decreased in cells reconstituted with GFP MRE11 M13 and GFP MRE11 M3 in contrast to GFP MRE11 WT and M1. Treatment with siMRE11 led to a further decrease of the expression of GFP MRE11 M13 and M3 (Figures 4d and e and Supplementary Figure S4g). As frequent transfection with siLUC lead to increased intensity of γH2AX staining in cells (Supplementary Figure S4f) and as R2TP complex may be important under stress conditions, we reasoned that decrease of GFP signal of M13 and M3 constructs after siLUC may be a result of stress caused by frequent transfection *per se* or due to constant DNA-damage stress caused by the transfection and that depletion of endogenous MRE11 may enhance this effect in MRE11 M3- and M13-reconstituted cells. Based on these results, we conclude that CK2-dependent interaction between MRE11 with PIH1D1 is important for MRE11 stability and that MRE11 serine S688/689 is the main site involved in this interaction.

Overall, we believe that we discovered a new mode of regulation of MRE11 stability by CK2 and the HSP90 cochaperone R2TP complex through its phospho-specific interaction with the R2TP subunit PIH1D1. As the R2TP complex is involved in assembly and/or quality control of large protein complexes, the interaction between MRE11 and PIH1D1 may be important for the assembly of complexes comprising MRE11 such as MRN, which is in accordance with the fact that ATLD1 human and mouse cells have overall decreased levels of all MRN complex components. The presence of serine 558/561 in the ATLD1 mutation may allow weaker interaction with PIH1D1 that would be sufficient to keep MRN complex level high enough for patient and mice survival.
