# Supplementary material for: MRE11 stability is regulated by CK2-dependent interaction with R2TP complex
Source: Oncogene. 2017 Apr 24;36(34):4943–50. doi: 10.1038/onc.2017.99 (PMC5531254; doi:10.1038/onc.2017.99)
Supplement: Supplementary Information [file onc201799x1.pdf]

Supplementary Information

MRE11 stability is regulated by CK2-dependent interaction with R2TP complex

Patrick von Morgen[1,2], Kamila Burdova[1], Thomas G. Flower[3], Nicola J. O'Reilly[4,] Simon J. Boulton[5], Stephen J. Smerdon[3], Libor Macurek[1,*] and Zuzana Hořejší[1,6,*]

Contains:

Supplementary Tables S2a and S2b

Supplementary Figures S3 and S4

Table S2a
Biotinylated peptides used in peptide pull-down assay

**Peptide**	**Sequence**
TEL2 S487/S491	Bio-eahx-IVDGGVPQAQLAGSDSDLDSDDEFVPYDMSGDREL
TEL2 pS487/pS491	Bio-eahx-IVDGGVPQAQLAGSDSDLDSDDEFVPYDMSGDREL
MRE11 S558/S561	Bio-eahx-MSIDLAEQMANDSDDSISAATN
MRE11 pS558/pS561	Bio-eahx-MSIDLAEQMANDpSDDpSISAATN
MRE11 pS558	Bio-eahx-MSIDLAEQMANDpSDDSISAATN
MRE11 pS561	Bio-eahx-MSIDLAEQMANDSDDpSISAATN
MRE11 S649	Bio-eahx-TKNYSEVIEVDESDVEEDIFPTTS
MRE11 pS649	Bio-eahx-TKNYSEVIEVDEpSDVEEDIFPTTS
MRE11 S688/S689	Bio-eahx-QVSKGVDFESSEDDDDDPFMNTSS
MRE11 pS688/pS689	Bio-eahx-QVSKGVDFEpSpSEDDDDDPFMNTSS
MRE11 pS688	Bio-eahx-QVSKGVDFEpSSEDDDDDPFMNTSS
MRE11 pS689	Bio-eahx-QVSKGVDFESpSEDDDDDPFMNTSS

Peptides were synthesized at The Francis Crick Institute Protein Chemistry Laboratory, eahx is a flexible linker.

Table S2 b ITC analysis

Protein	Peptide	Sequence	K_d (μM)	ΔH^o (kcal mol^{-1})	$-T\Delta S^o$ (kcal mol^{-1})	N
PIH1D1 (51-180)	TEL2 pS491	487- YSDLDpSDDEFVPY	9.8±1.0	-4.1±0.6	9.0±2.2	1.3±0.1
PIH1D1 (51-180)	MRE11 pS558/pS561	554 - YMANDpSDDpSISAA	26.1±1.8	-6.7±0.3	-2.0±1.0	1.1±0.0
PIH1D1 (51-180)	MRE11 pS558	554 - YMANDpSDDSISAA	ND	ND	ND	ND
PIH1D1 (51-180)	MRE11 pS561	554 - YMANDSDDpSISAA	ND	ND	ND	ND
PIH1D1 (51-180)	MRE11 pS688/pS689	685 - YDFEpSpSEDDDDD	40.9±5.9	-4.2±0.3	6.1±0.7	0.8±0.0
PIH1D1 (51-180)	MRE11 pS688	685 - YDFEpSSEDDDDD	ND	ND	ND	ND
PIH1D1 (51-180)	MRE11 pS689	685 - YDFESpSEDDDDD	ND	ND	ND	ND

Numbers preceding the peptide sequences correspond to the position of the first amino acid in the protein sequence. The PIH1D1 binding motif is underscored with pS indicating phosphoserine. An N-terminal tyrosine residue was included for concentration determination. Experiments were performed in in triplicate, with data shown as means ± standard deviation. Not defined (ND) interaction indicates weak and/or constant signal under the condition used (~80μM protein and ~800μM peptide).

Figure S3

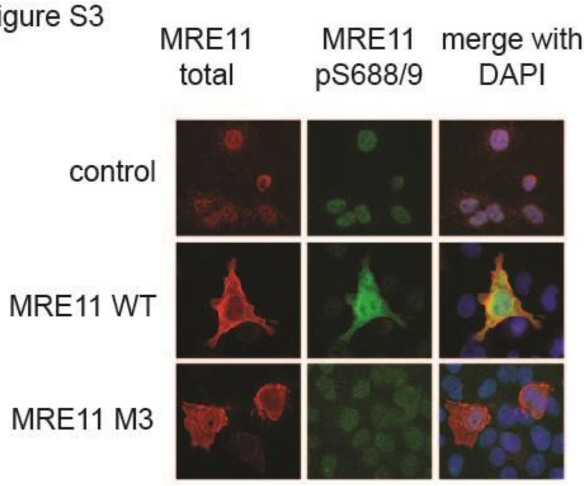

Supplementary Figure 3

Immunofluorescence staining of FLAG MRE11 WT and M3 with MRE11 total and phospho-serine 688/689 antibody. The cells were grown on coverslips, fixed with 4% paraformaldehyde for 15 min at room temperature (rt), permeabilized with 0.2 % Triton-X100 for 5 min at rt and stained with total MRE11 antibody (GeneTex, GTX70212, 1:200) and antibody recognizing MRE11 phospho-serine 688/689 (1:200) one hour at rt.

Figure S4

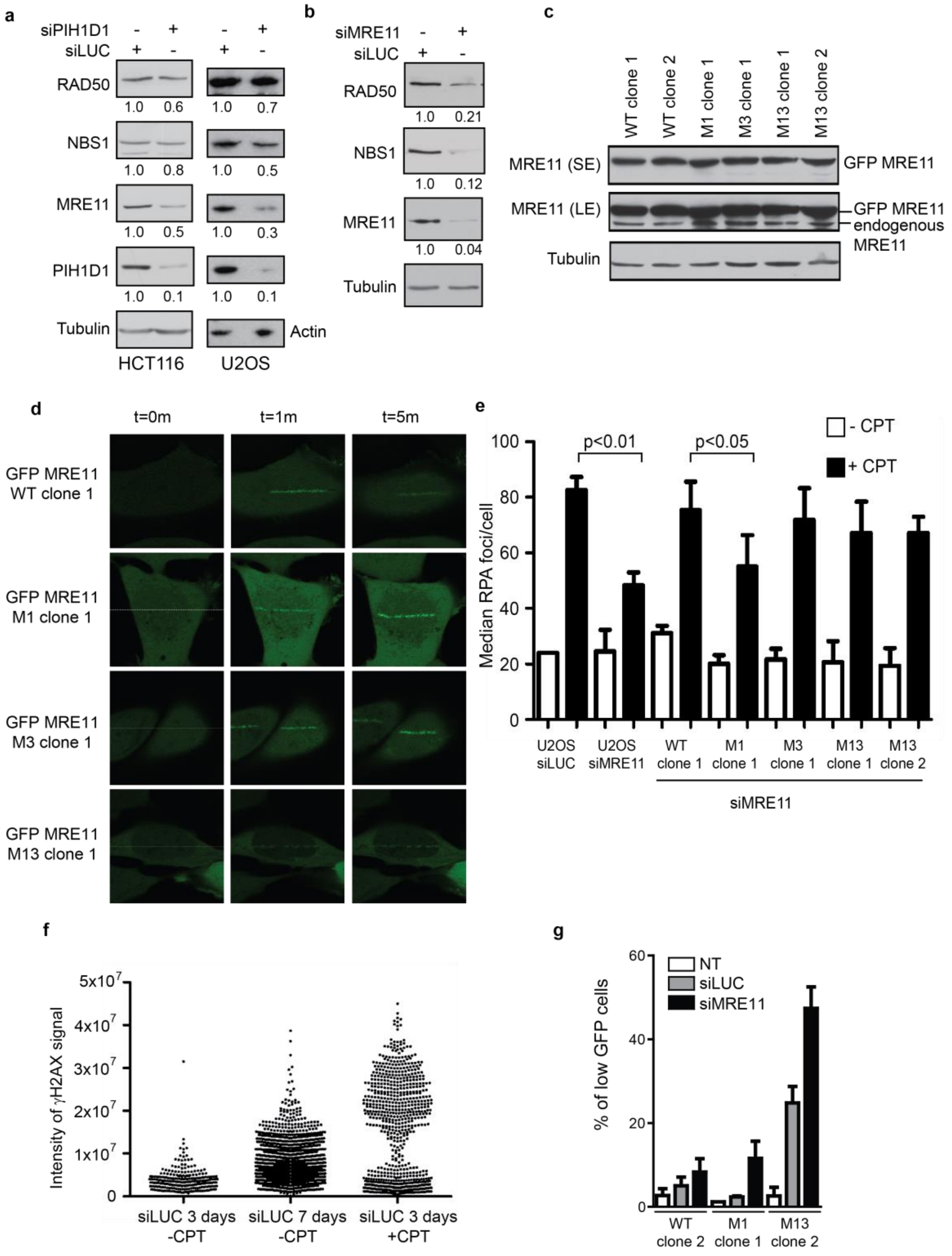

Supplementary Figure 4

(**a**) Down-regulation of PIH1D1 leads to decreased levels of MRE11 in HCT116 and U2OS cells. HCT116 and U2OS cells were treated with siLuc or siPIH1D1 every 48 hours for 10 and 6 days, respectively, before western blot analysis using indicated antibodies. Signal was quantified with ImageJ and normalized

to the tubulin or actin and to the signal measured in siLUC control. Representative result from three independent experiments is shown. (**b**) Knock-down of MRE11 leads to decreased levels of NBS1 and RAD50. U2OS cells were treated with siMRE11 every 48 hours for 5 days before western blot analysis. (**c**) Comparison of GFP-MRE11 expression in U2OS cells stably transfected with pcDNA4/TO GFP MRE11. Following 4 weeks selection with Zeocin, individual clones were expanded and analysed by western blotting. (**d**) Recruitment of the GFP MRE11 to the DNA damage sites. Cells were grown on a μ-Slide 8-well (Ibidi). Prior to imaging media was replaced with Leibovitz's L-15 medium (Invitrogen). Cells were imaged in a temperature-controlled (37°C) chamber using Leica SP5 DMI 6000 confocal microscope equipped with 63×/1.40 objective (HC PL APO CS2 OIL; Leica). DNA damage was induced by microirradiation of a narrow region of interest with a 405 nm laser (CUBE405-100C, 100 mW 100% power) and cells were imaged every 30 seconds for 5 minutes. Representative images are shown. (**e**) Quantification of RPA foci after camptothecin treatment of the GFP MRE11 stable clones. U2OS cells grown on coverslips were treated for 12 days with siMRE11 or siLuc. Prior to 1 h treatment with 0.5μM camptothecin (CPT) cells were incubated for 15 minutes with 10 μM EdU. Cells were preextracted for 5 min 4°C with preextraction buffer (25mM Hepes pH 7.4, 50 mM NaCl, 1 mM EDTA, 3 mM $MgCl_2$, 0.3 M sucrose, 0.5 % Triton X-100), fixed for 15 min rt with 4 % paraformaldehyde and blocked with 3 % BSA for 30 min rt. Click-iT reaction was performed with Alexa fluor azide 647 according to manufactures protocol (Invitrogen). Next, the cells were incubated for 2 h with RPA antibody at rt (Abcam, ab2175) and for 1 hr at rt with secondary antibody (Alexa Fluor 568). RPA foci number was analysed on an automated high-content screening station (Scan^R; IX81 Olympus; ORCA-285 camera) equipped with a 40X/1.3 NA objective (RMS40X-PFO; Olympus). DAPI signal was used to identify the nuclei, EdU staining was used to identify the S phase cells and the number of RPA foci per nucleus was determined using a spot detection module. Median amount of RPA foci per S phase cell was compared using the Graphpad prism software (n=3). Two-tailed paired t-test was used to compare the different treatments and the rescue with WT or mutant MRE11. (**f**) Repeated siRNA

treatment induces DNA damage. U2OS cells were grown on coverslips and treated for 7 days with siLuc, treated for 1 hour with 1 μM camptothecin (CPT), preextracted for 5 min 4ºC with preextraction buffer, fixed for 15 min rt with 4% paraformaldehyde, blocked with 3% BSA for 30 min rt, incubated for 2 hr rt with γH2AX antibody (Milipore, 05-636) and 1 hour rt with secondary antibody (Alexa Fluor 568). Total intensity of the γH2AX signal was determined per nucleus using Scan^R; IX81 Olympus with 40X/1.3 NA objective. (**g**) Mutation of serine 558/561 together with 688/69 leads to destabilization of MRE11. Individual clones of U2OS cells stably transfected with GFP MRE11 WT, M1, and M13 were transfected with siRNA targeting luciferase (siLUC), 3'UTR of MRE11 (siMRE11) or left untreated. siRNA was retransfected every 2 days and cells were grown for 17 days. Cells were collected and incubated with Hoechst 33258. GFP signal was analysed in Hoechst negative cells by flow cytometry using BD LSR II. The percentage of cells that have low GFP MRE11 was quantified using Graphpad prism software (n=3). Bars indicate mean plus SD.
